# Identification and Validation of a Novel Major Quantitative Trait Locus for Plant Height in Common Wheat (*Triticum aestivum* L.)

**DOI:** 10.3389/fgene.2020.602495

**Published:** 2020-10-22

**Authors:** Zhiqiang Wang, Haiyan Hu, Xiaojun Jiang, Yang Tao, Yu Lin, Fangkun Wu, Shuai Hou, Shihang Liu, Caixia Li, Guangdeng Chen, Yaxi Liu

**Affiliations:** ^1^Triticeae Research Institute, Sichuan Agricultural University, Chengdu, China; ^2^School of Life Sciences and Technology, Henan Institute of Science and Technology, Xinxiang, China; ^3^Rice Research Institute, Sichuan Agricultural University, Chengdu, China; ^4^College of Resources, Sichuan Agricultural University, Chengdu, China; ^5^State Key Laboratory of Crop Gene Exploration and Utilization in Southwest China, Chengdu, China

**Keywords:** wheat, plant height, quantitative trait locus, validation, candidate gene

## Abstract

Plant height (PH) plays a pivotal role in plant morphological architecture and is associated with yield potential in wheat. For the quantitative trait locus (QTL) analysis, a recombinant inbred line population was developed between varieties differing significantly in PH. Two major QTL were identified on chromosomes 4B (*QPh.sicau-4B*) and 6D (*QPh.sicau-6D*) in multiple environments, which were then validated in two different backgrounds by using closely linked markers. *QPh.sicau-4B* explained 10.1–21.3% of the phenotypic variance, and the location corresponded to the dwarfing gene *Rht-B1*. *QPh.sicau-6D* might be a novel QTL for PH, explaining 6.6–13.6% of the phenotypic variance and affecting spike length, thousand-kernel weight, and spikelet compactness. Three candidate genes associated with plant growth and development were identified in the physical interval of *QPh.sicau-6D*. Collectively, we identified a novel stable and major PH QTL, *QPh.sicau-6D*, which could aid in the development of closely linked markers for marker-assisted breeding and cloning genes underlying this QTL.

## Introduction

Bread wheat (*Triticum aestivum* L.) is an important staple crop, ranking the third after maize and rice in terms of yield in China (Edae et al., 2014; Liu et al., 2018). According to the Food and Agriculture Organization of the United Nations^1^, the global wheat grain yield in 2017 was 771.7 million tons, contributing to approximately 20% of the calories consumed by humans. Plant height (PH) is an important yield component trait associated with plant morphological architecture and other yield-related traits, such as spike length, spikelet number per spike, spikelet compactness (SC), and thousand-kernel weight (TKW), thus affecting the yield potential (Sakamoto and Matsuoka, 2004; Gao et al., 2015; Kowalsk et al., 2016; Guan et al., 2018). To develop high grain yield lines, Donald (1968) proposed the idea of breeding crop ideotypes with a relatively short PH, single culm, strong stem, and large and erect ear.

In view of the importance of PH in wheat yield, it is imperative to identify more candidate genes responsible for PH from wheat germplasm resources. In the past two decades, numerous major and minor QTL influencing PH have been identified on 21 chromosomes in wheat, and some of them have been applied in wheat breeding (Peng et al., 1999; Liu et al., 2002; Griffiths et al., 2012; Würschum et al., 2015; Tian et al., 2017; Hassan et al., 2019). Additionally, several PH genes have been cloned, such as *Rht-B1* and *Rht-D1* (located on chromosome 4B and 4D, respectively), and highly adopted in breeding practices during the green revolution; they encode the DELLA proteins, participating in gibberellin signaling, and thereby affecting PH (Peng et al., 1999; Pearce et al., 2011). *Rht18* encodes a gibberellic acid (GA) 2-oxidase protein, which regulates the balance of GA intermediates and inactive GA, leading to a semi-dwarf phenotype in wheat (Ford et al., 2018). In *Arabidopsis*, extensively studied dwarf mutants such as the *yda* and *pat10* mutants, which are defective in growth and development, have been shown to significantly differ from the wild-type plants in terms of PH (Lukowitz et al., 2004; Zhou et al., 2013).

In this study, a recombinant inbred lines (RILs) population was used for QTL mapping of PH with a genetic map using the 90K SNP array and phenotyping in six environments to identify major QTL for PH. The effects of the major QTL for PH were further assessed in different genetic backgrounds.

## Materials and Methods

### Plant Materials

Three populations of RILs were generated by single-seed descent in the field in Sichuan Agricultural University, Wenjiang (103°51′E, 30°43′N), with H461 as a common parent. These three populations were as follows: H461/CN16 (HCN; 249 F_8_ lines), H461/CM107 (HCM; 200 F_7_ lines), and H461/MM37 (HMM; 142 F_6_ lines).

The HCN population was used for QTL mapping, whereas the other populations (HCM and HMM) were used for validating the major QTL identified in the HCN population.

### Phenotypic Evaluation

The three populations were planted in six different environments for phenotypic evaluation: Wenjiang in 2015 and 2019 (2015WJ and 2019WJ); Chongzhou (103°38′E, 30°32′N) in 2015, 2017, and 2019 (2015CZ, 2017CZ, and 2019CZ); and Ya’an (103°0′E, 29°58′N) in 2015 (2015YA). Each plot consisted of three rows, with a length of 1.5 m and an inter-row spacing of 30 cm; the sowing density was 15 seeds per row. For each plot, five plants were randomly chosen to measure PH, from the plant base to the tip of the spike, and calculate the mean PH. The main spike of five plants were selected to measure the spikelet number per spike (SN) and spike length (SL). The TKW was measured using an electronic balance with three replications. Flowering time (FT) was recorded as the date when half of the plants in each plot flowered after sowing. The SC was calculated by dividing the SL by the SN.

Analysis of variance (ANOVA) and calculation of Pearson’s correlation coefficients among different environments were performed using SPSS 22 (IBM SPSS, Armonk, NY, United States). Frequency distribution was processed using MS Excel, and the best linear unbiased prediction (BLUP) for target traits was calculated using R version 3.5.2 (Team, 2013). Broad-sense heritability (*h*^2^) was calculated across environments as described by Smith et al. (1998). The correlations between PH and the factors SN, SL, SC, TKW, and FT were calculated based on the BLUP values, and Student’s *t*-test was performed to determine significant differences between two groups using SPSS 22.

### QTL Mapping

The HCN population was used for constructing a whole-genome genetic linkage map using the 90K SNP array (Wang et al., 2016) for QTL mapping, consisting of 7808 SNP polymorphic markers in parents distributed in 50 linkage groups and covers a total genetic distance of 3486.44 cM, with an average distance of 0.45 cM between the adjacent markers.

MapQTL 6.0 (Van Ooijen and Kyazma, 2009) was used for the QTL analysis. Kruskal–Wallis test was used to evaluate the degree of association between markers and PH. Interval mapping (IM) was then used to identify major QTL and markers significantly associated with PH. For each trial, a test of 1000 permutations was performed to identify the LOD threshold corresponding to a genome-wide false discovery rate of 1%. Based on the permutation test, threshold LOD values between 2.4 and 3.3 were used to confirm the presence of a QTL. The QTL were named based on the International Rules of Genetic Nomenclature^2^. “Ph” and “sicau” stand for “plant height” and “Sichuan Agricultural University,” respectively.

### Validation of the Major QTL

The flanking markers of the major QTL were mapped to the physical map of the wheat cultivar Chinese Spring (IWGSC RefSeq v1.0), and the sequence information in the QTL interval was obtained. To develop Kompetitive allele specific PCR (KASP) markers closely linked to the QTL, the partial sequence information of the QTL interval was amplified in CN16 and H461 by PCR to search for polymorphic sites. The newly developed KASP markers were remapped into the genetic map.

The markers closely linked to the QTL were used for identifying alleles in different genetic backgrounds (populations HCM and CMM). The lines were classified into two groups: genotypes with homozygous alleles from H461 (designated AA) and those with homozygous alleles from alternative parents (designated BB). The mean PH from homozygotes was used for measuring the QTL effects, and Student’s *t*-test was used to determine the significance of differences between the two groups in each population.

### Predicted Candidate Genes

The gene information of the QTL interval was obtained from IWGSC RefSeq v1.1 annotation. Expression values as transcripts per million (TPM) were obtained from the expVIP Wheat Expression Browser^3^ (Borrill et al., 2016), genes with a low expression (TPM < 0.5) in various tissues were excluded and the mean expression values were visualized by TBtools (Chen et al., 2020). The remaining genes were annotated by KOBAS v3.0 (Ai and Kong, 2018) BLAST against the corresponding protein sequences in rice and *Arabidopsis thaliana*. The genomic DNA of parents was extracted from the leaf samples using the Plant Genomic DNA kit (Biotechnologies, CA) and used to amplify candidate genes for sequence analysis.

## Results

### Phenotyping of the HCN Population

In different environments, the PH of H461 ranged from 79.00 to 88.22 cm, and that of CN16 ranged from 61.60 to 76.75 cm. Moreover, significant differences in PH were observed between H461 and CN16 (Table 1). The frequency of PH in the HCN population showed continuous distribution, ranging from 51.50 to 124.2 cm (Table 1 and Supplementary Figure S1); this implied that PH was affected by multiple loci. The *h*^2^ of PH was 0.83, and Pearson’s correlation coefficients between the different environments ranged from 0.232 to 0.872 (*P* < 0.01; Table 2).

**TABLE 1:** Phenotypic variation of the mapping population H461 × CN16 and parental lines in different environments.

**Trait**	**Environment**	**Parents**		**Population**			
			
		**H461**	**CN16**	**Range**	**Mean**	**SD**	***h*^2^**
	2015WJ	79.00**	61.60	64.32–115.84	84.72	8.08	
	2015YA	82.80**	74.02	62.47–114.44	83.25	8.87	
PH	2015CZ	85.76**	75.20	64.42–124.20	87.45	8.89	
	2017CZ	85.13**	76.75	55.33–111.17	79.73	9.14	
	2019WJ	86.94**	70.61	51.50–109.00	78.60	8.36	
	2019CZ	88.22**	76.17	58.10–110.78	81.10	7.80	
	BLUP	82.33	73.75	63.01–105.93	80.53	5.71	0.83
SN	BLUP	22.50	19.71	19.75–23.55	21.30	0.55	0.65
SL	BLUP	14.06	10.92	10.41–13.95	12.13	0.56	0.72
SC	BLUP	1.60	1.80	1.51–1.99	1.77	0.09	0.80
TKW	BLUP	52.49	44.66	38.99–58.31	49.41	3.35	0.81
FT	BLUP	142.06	141.10	137.78–149.16	141.75	2.12	0.84

SD, standard deviation; *h*^2^, broad-sense heritability; BLUP, phenotype values based on BLUP. **indicates significant differences at *P* < 0.01.

**TABLE 2:** Correlation coefficients for plant height (PH) in the HCN population evaluated in different environments.

	**2015WJ**	**2015YA**	**2015CZ**	**2017CZ**	**2019WJ**
2015YA	0.334**				
2015CZ	0.549**	0.661**			
2017CZ	0.613**	0.242**	0.444**		
2019WJ	0.570**	0.232**	0.405**	0.633**	
2019CZ	0.632**	0.314**	0.477**	0.644**	0.872**

**indicates significant differences at *P* < 0.01.

The BLUP values of PH, SN, SL, SC, TKW, and FT are shown in Table 1. Phenotypic correlation coefficients between PH and other spike-related traits were obtained based on the BLUP values (Table 3). PH was highly significantly correlated with the TKW (*P* < 0.01) and significantly correlated with the SN and SL (*P* < 0.05). No significant correlation was observed between PH and SC or FT (Table 3).

**TABLE 3:** Correlation coefficients among the BLUP value for plant height (PH) with spikelet number per spike (SN), spike length (SL), spikelet compactness (SC), thousand kernel weight (TKW) and flowering time (FT).

**Trait**	**PH**
SN	0.180*
SL	0.182*
SC	−0.075
TKW	0.249**
FT	0.073

**and *indicate significant correlations at *P* < 0.05 and *P* < 0.01, respectively.

### Identification of QTL for PH

Three QTL for PH were identified using the IM analysis (Table 4). The first QTL (*QPh.sicau-4B*) was located on the short arm of chromosome 4B, between the markers Tdurum_contig64772_417 and Excalibur_rep_c113261_400. *QPh.sicau-4B* was a stable major QTL with the additive effects from H461, and it explained 10.1–21.3% of the phenotypic variance, with LOD values ranging from 4.15 to 9.39. It was identified in five environments and the combined analysis (BLUP). The second QTL (*QPh.sicau-6D*) was located on the short arm of chromosome 6D, between the markers IACX10982 and BS00063175_51. *QPh.sicau-6D* was a stable major QTL with the additive effects from H461; it explained 6.6%–13.6% of the phenotypic variance, with LOD values ranging from 2.67 to 5.80, identified in all environments and using BLUP. The third QTL (*QPh.sicau-3B*) was located on 3B, identified only in 2017CZ, and it explained 7.9% of the phenotypic variance.

**TABLE 4:** Quantitative trait loci (QTL) for plant height identified in the H461 × CN16 recombinant inbred line population evaluated in different environments.

**QTL**	**Environment**	**Interval (cM)**	**Flanking markers**	**LOD**	**PVE (%)^a^**	**Add^b^**
*QPh.sicau-4B*	2015WJ	94.00∼102.26	Tdurum_contig64772_417 and BS00023766_51	9.39	21.3	3.79
	2015CZ	94.00∼101.67	Tdurum_contig64772_417 and Excalibur_rep_c113261_400	7.76	17.9	3.97
	2015YA	94.00∼101.67	Tdurum_contig64772_417 and Excalibur_rep_c113261_400	5.19	12.7	3.35
	2019WJ	94.00∼102.26	Tdurum_contig64772_417 and BS00023766_51	4.15	10.1	2.70
	2019CZ	94.00∼102.26	Tdurum_contig64772_417 and BS00023766_51	4.84	11.6	2.70
	BLUP	94.00∼101.67	Tdurum_contig64772_417 and Excalibur_rep_c113261_400	6.19	14.4	2.28
*QPh.sicau-6D*	2015WJ	25.02∼31.43	Kukri_c34967_226 and BS00063175_51	3.97	9.6	2.54
	2015CZ	25.02∼31.43	Kukri_c34967_226 and BS00063175_51	3.49	8.5	2.64
	2015YA	28.53∼31.43	IACX10982 and BS00063175_51	3.30	8.3	2.58
	2017CZ	25.02∼31.43	Kukri_c34967_226 and BS00063175_51	5.71	13.4	3.37
	2019WJ	25.02∼31.43	Kukri_c34967_226 and BS00063175_51	2.84	7.0	2.24
	2019CZ	25.02∼31.43	Kukri_c34967_226 and BS00063175_51	2.67	6.6	2.03
	BLUP	28.53∼31.43	IACX10982 and BS00063175_51	5.80	13.6	2.13
*QPh.sicau-3B*	2017CZ	96.74∼98.46	BS00099633_51 and Kukri_c6907_80	3.27	7.9	2.65

*^a^ Percentage of the phenotypic variation explained. ^b^ Additive effect.*

### Effects of the Two Major PH QTL on PH and Other Panicle Traits

To identify the effect of the two major PH QTL (*QPh.sicau-4B* and *QPh.sicau-6D*) for other panicle traits, the BLUP values across six environments were used. For *QPh.sicau-4B*, lines with homozygous alleles from H461 and lines with homozygous alleles from CN16, classified into two groups, showed a significant difference (*P* < 0.05) for FT (Supplementary Figure S2). For *QPh.sicau-6D*, lines with homozygous alleles from H461 and those with homozygous alleles from CN16, classified into two groups, showed significant differences (*P* < 0.05) for SL, SC, and TKW (Supplementary Figure S3).

**FIGURE 1:**
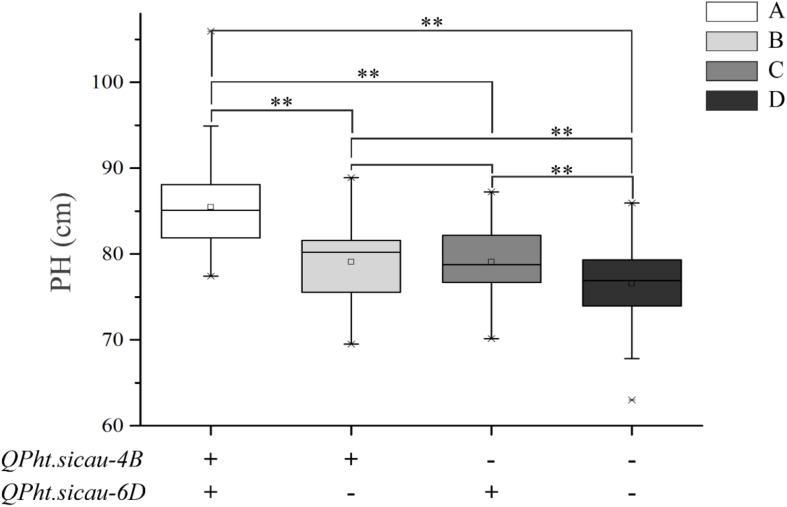
Effects of the *QPh.sicau-4B* and *QPh.sicau-6D* on PH in HCN population (A: carrying both the additive alleles of the two major QTL; B: only carrying the additive allele of *QPh.sicau-4B*; C: only carrying the additive allele of *QPh.sicau-6D*; and D: not carrying the additive alleles of the two major QTL). **indicates significant differences at *P* < 0.01.

For PH, the HCN population could be divided into the following four groups based on markers: (A) carrying both the additive alleles of two major QTL, (B) only carrying the additive allele of *QPh.sicau-4B*, (C) only carrying the additive allele of *QPh.sicau-6D*, and (D) not carrying the additive alleles of *QPh.sicau-4B* and *QPh.sicau-6D*. Comparative analyses among the four groups showed that the group A had the highest effect on PH, which was significantly higher than that of the groups B, C, and D. Furthermore, the groups B and C had significantly higher effects than that of the group D. Thus, *QPh.sicau-4B* and *QPh.sicau-6D* might significantly affect PH, with both having a significant effects on PH (Figure 1).

### Validation of QTL in Different Genetic Backgrounds

**FIGURE 2:**
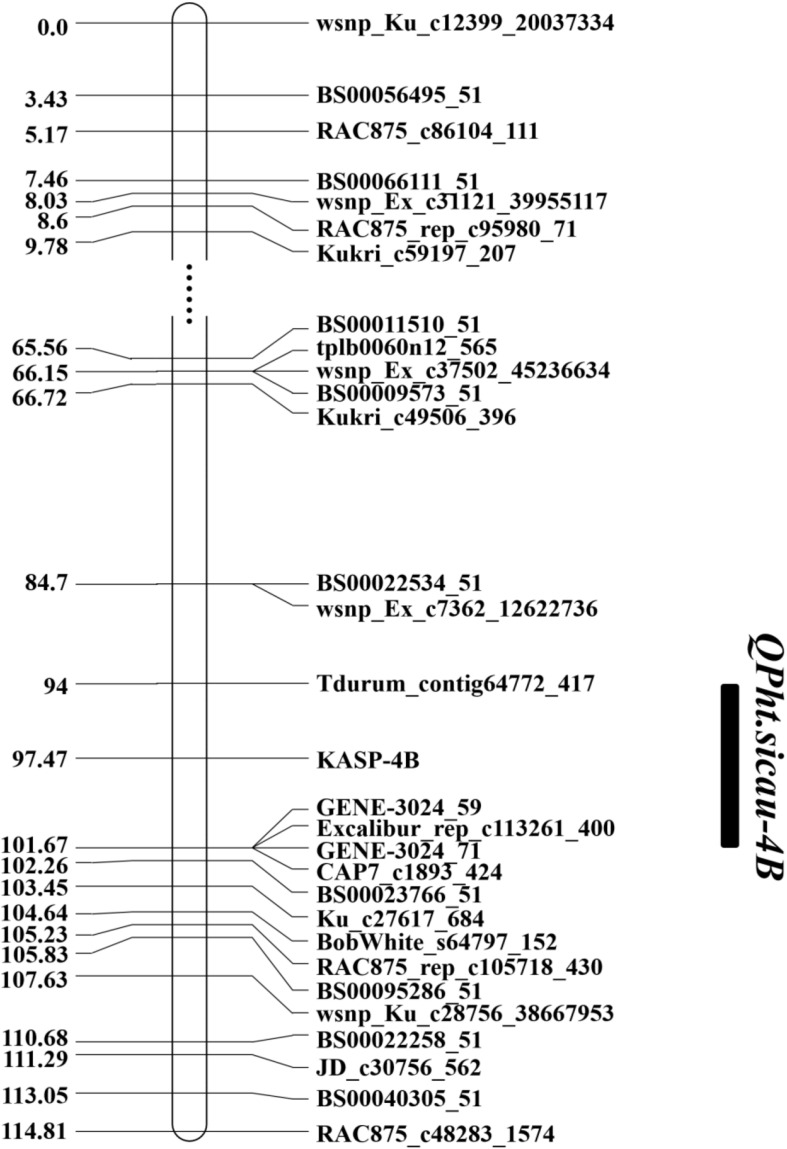
Genetic map of the major QTL *QPh.sicau-4B* with the marker KASP-4B.

**FIGURE 3:**
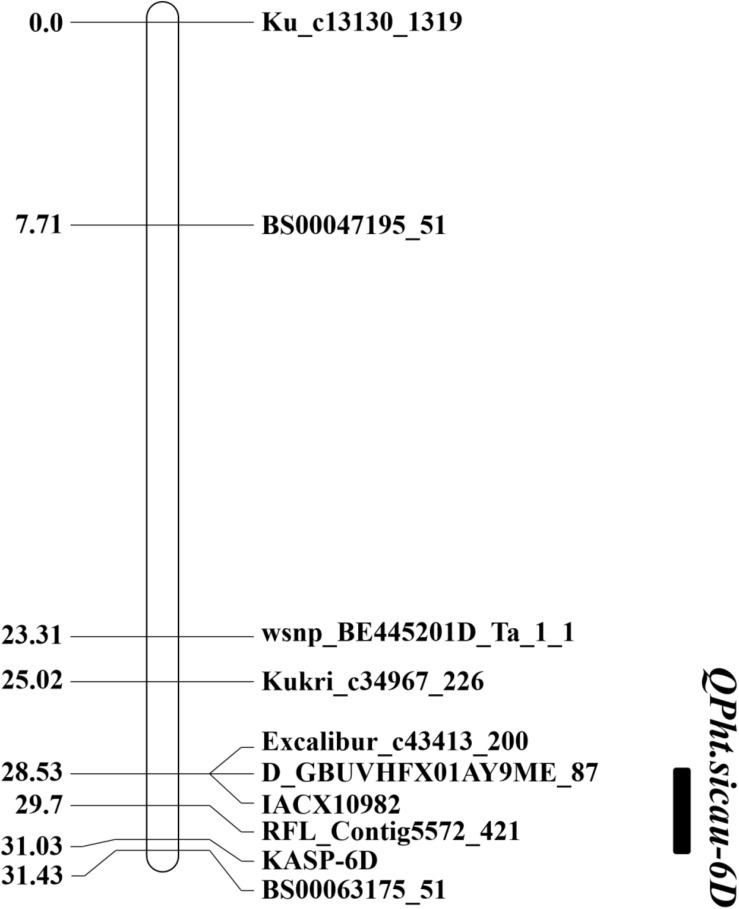
Genetic map of the major QTL *QPh.sicau-6D* with the marker KASP-6D.

Based on the QTL mapping results and Sanger sequencing of the PCR products of H461 and CN16, two KASP markers (KASP-4B and KASP-6D, Supplementary Table S1) were developed and used to reconstruct the genetic map. The KASP-4B marker was found to be closely linked to *QPh.sicau-4B*, whereas the KASP-6D marker was closely linked to *QPh.sicau-6D* (Figures 2,3).

Two populations (HCM and HMM) were used for evaluating the effects of the two major QTL in different genetic backgrounds, and the KASP markers were used to identify the genotype. For *QPh.sicau-4B*, KASP-4B was used to identify the alleles in the HCM and HMM populations and were classified into two groups. Significant differences (*P* < 0.05) were detected between “AA” and “BB” genotypes in three environments for HCM and four environments for HMM (Table 5). The differences in PH ranged from 2.56 to 9.24% in the HCM and HMM populations. For *QPh.sicau-6D*, KASP-6D was used to identify the alleles in the HCM and HMM populations and were classified into two groups. Significant differences (*P* < 0.05) were detected between “AA” and “BB” genotypes in four environments for HCM and four environments for HMM (Table 6). The differences in PH ranged from 3.77 to 12.41% in the HCM and HMM populations. And the effects of Qph.sicau-6D was higher than Qph.sicau-4B in the validation populations, which may be responsible by different genetic backgrounds.

**TABLE 5:** Effects of *QPh.sicau-4B* in two validation populations.

**Population**	**Environment**	**Parent^a^**		**AA^b^**	**BB^c^**	**Difference**	***P*-value**
		**Parent1**	**Parent2**				
HCM	2015YA	82.80	76.25	92.50	89.43	3.43%*	<0.05
HCM	2015WJ	79.00	71.19	93.07	90.22	3.16%*	<0.05
HCM	2015CZ	85.76	73.22	94.63	90.61	4.44%**	<0.01
HCM	2017CZ	85.13	84.44	86.25	84.03	2.64%	0.09
HCM	BLUP	82.33	72.3	89.58	87.34	2.56%*	<0.05
HMM	2015WJ	79.00	58.36	84.29	77.42	8.87%**	<0.01
HMM	2017CZ	85.13	80.33	86.80	79.46	9.24%**	<0.01
HMM	2019WJ	86.94	70.83	75.82	70.79	7.11%*	<0.05
HMM	2019CZ	88.22	56.25	82.67	76.21	8.48%**	<0.01
HMM	BLUP	82.33	70.53	82.09	76.14	7.81%**	<0.01

**and *indicate extremely significant difference and significant difference, respectively. ^a^“Parent1” represents H461, “Parent2” represents CM107 or MM37. ^b^“AA” represents homozygous alleles from H461. ^c^“BB” represents homozygous alleles from non-H461 parents.

**TABLE 6:** Effects of *QPh.sicau-6D* in two validation populations.

**Population**	**Environment**	**Parent^a^**		**AA^b^**	**BB^c^**	**Difference**	***P*-value**
		**Parent1**	**Parent2**				
HCM	2015YA	82.80	76.25	93.92	89.27	5.21%**	<0.01
HCM	2015WJ	79.00	71.19	93.19	88.45	5.36%**	<0.01
HCM	2015CZ	85.76	73.22	94.76	89.61	5.75%**	<0.01
HCM	2017CZ	85.13	84.44	86.78	83.56	3.85%**	0.01
HCM	BLUP	82.33	72.3	90.01	86.74	3.77%**	<0.01
HMM	2015WJ	79.00	58.36	85.95	77.25	11.26%**	<0.01
HMM	2017CZ	85.13	80.33	89.50	79.62	12.41%**	<0.01
HMM	2019WJ	86.94	70.83	76.25	71.29	6.96%*	<0.05
HMM	2019CZ	88.22	56.25	83.02	76.70	8.24%**	<0.01
HMM	BLUP	82.33	70.53	82.96	76.36	8.64%**	<0.01

**and *indicate extremely significant difference and significant difference, respectively. ^a^“Parent1” represents H461, “Parent2” represents CM107 or MM37. ^b^“AA” represents homozygous alleles from H461. ^c^“BB” represents homozygous alleles from non-H461 parents.

### Potential Candidate Genes

**FIGURE 4:**
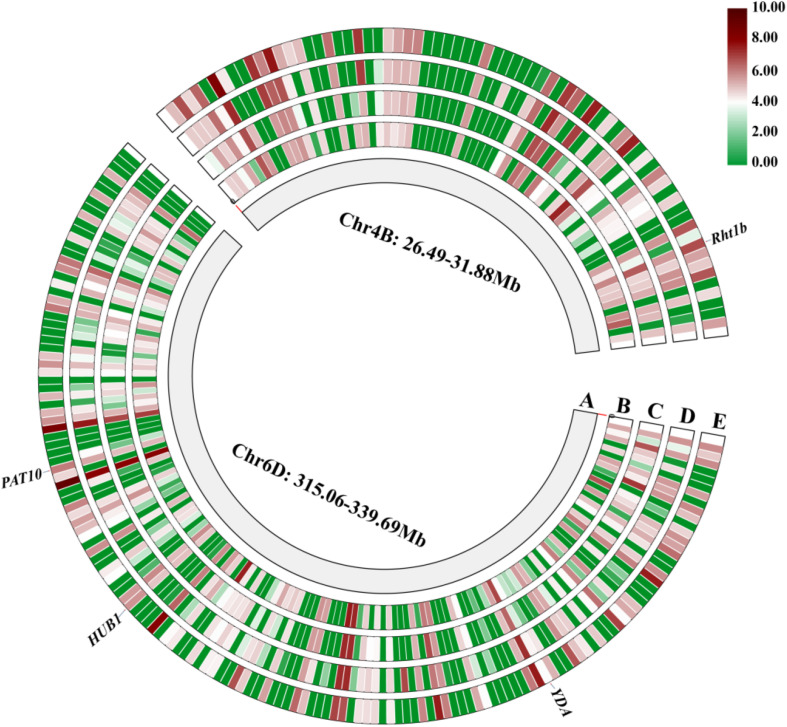
The genes expression of in various tissues in the *QPh.sicau-4B* interval and *QPh.sicau-6D* interval from the expVIP Wheat Expression Brower (A: physical map of chromosome 4B and chromosome 6D, B: root, C: leaf, D: spike, E: grain).

A total of 224 high-confidence (HC) genes were selected from the *QPh.sicau-4B* and *QPh.sicau-6D* intervals, and 62 HC genes with low expression in various tissues were excluded (Figure 4). Finally, 47 HC genes from *QPh.sicau-4B* were selected for gene annotation, including *Rht-B1*. And 115 HC genes from *QPh.sicau-6D* were selected for gene annotation, including *YDA*, *BUB1*, and *PAT10* (Supplementary Table S2 and Table 7).

**TABLE 7:** The information of the candidate genes.

**Gene ID**	**Gene name**
TraesCS4B02G043100	*Rht1b*
TraesCS6D02G227300	*YDA*
TraesCS6D02G233000	*HUB1*
TraesCS6D02G234900	*PAT10*

**FIGURE 5:**
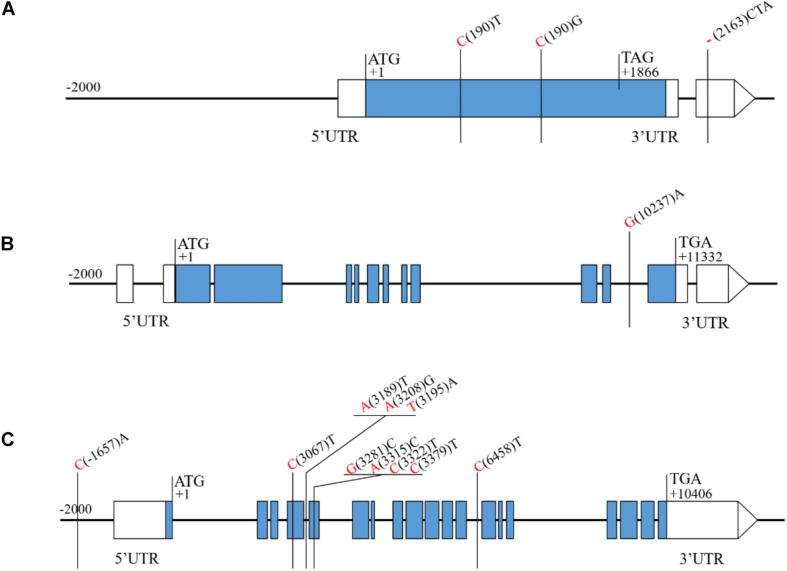
Sequence analysis of the candidate genes showing the SNPs and Indels between H461 and CN16. The nucleotide of H461 and CN16 are shown in red and black, respectively (**A:** *Rht-1B*, **B:** *TraesCS6D02G227300*, **C:** *TraesCS6D02G233000*).

Sequence analysis of the four candidate genes, revealed 2 SNPs and 1 insertion/deletion (indel) between H461 and CN16 for *Rht-B1*, one T for C substitution (C(190)T) converts the codon (CGA) to a translational stop codon (TGA) in CN16 (Figure 5A). For *TraesCS6D02G227300*, 1 SNP in the intron region between H461 and CN16 was found (Figure 5B). Five SNPs in the coding sequence, 4 SNPs in the intron region, and 1 SNP in the promoter region for *TraesCS6D02G233000* were detected between H461 and CN16 (Figure 5C). However, no sequence variation between H461 and CN16 was identified for *TraesCS6D02G234900*.

## Discussion

Plant height is a critical trait that influences plant architecture and grain yield potential in wheat, and it is controlled by multiple genes functioning together (Spielmeyer et al., 2007; Singh et al., 2016). Exploring PH QTL and genes is essential for wheat breeding, and the identification of QTL associated with PH on different chromosomes has been widely reported (Zhang et al., 2010; Liu et al., 2014; Gao et al., 2015; Chai et al., 2018). In this study, two stable and major QTL for PH were identified in different environments and were validated in different genetic backgrounds. *QPh.sicau-4B* was located in a 7.67-cM interval and mapped between 26.49 and 31.88 Mb on the physical map of chromosome 4B (Figures 2, 4). *QPh.sicau-6D* was located in a 2.9-cM interval and mapped between 315.06 and 339.69 Mb on the physical map of chromosome 6D (Figures 3, 4).

### *QPh.sicau-6D* Is a Novel QTL for PH

*QPh.sicau-6D* was physically mapped between 315.06 and 339.69 Mb of chromosome 6D. Several known QTL responsible for PH have been mapped on chromosome 6D, including *QPh.spa-6D*, and *QPh.cau-6D* (Supplementary Table S3). We tried to compare the physical positions of these QTL to assess their relationship with *QPh.sicau-6D*. Physical mapping showed that *QPh.spa-6D* (Singh et al., 2016) was mapped to intervals 3.6–5.8 Mb of the chromosome 6D, was far away from *QPh.sicau-6D*, indicating that they were different loci. The confidence interval of *QPh.cau-6D* (Guan et al., 2018) was mapped between 283.97 and 292.07 Mb on the physical map. No physical interval of *QPh.cau-6D* overlapped with *QPh.sicau-6D*, verifying that they were different loci. These comparisons implied that *QPh.sicau-6D* was likely a novel QTL for PH.

*QPh.cau-4B.2* (Guan et al., 2018) and *QPH.caas-4BS.2* (Gao et al., 2015) were mapped to intervals 29.0–35.5 Mb and 21.4–46.6 Mb, respectively, in the 4B chromosome physical map (Supplementary Table S3). Furthermore, the two QTL overlapped with *QPh.sicau-4B*, implying that *QPh.sicau-4B* was likely the same locus as *QPh.cau-4B.2* and *QPH.caas-4BS.2*.

### Correlations Between the Major PH QTL and Other Spike-Related Traits

Gao et al. (2015) and Guan et al. (2018) reported that PH was significantly positively correlated with the TKW but not with the other spike-related traits. Zhai et al. (2016) reported that PH was positively correlated with the SL but negatively correlated with the SN and SC. In this study, Pearson’s correlation analysis showed that PH was positively correlated with the SL, SN, and TKW (Table 3). This might be because of lines carrying different PH QTL that affect the correlation of PH with other traits. Further analysis of QTL responsible for significant differences in the SL, SC, and TKW between lines with different alleles at *QPh.sicau-6D* (Supplementary Figure S3) suggested that *QPh.sicau-6D* confers pleiotropic effects on the SL, TKW, and SC. This interesting perspective warrants further investigation. Additionally, *QPh.sicau-4B* and *QPh.sicau-6D* demonstrated superimposed effects on PH (Figure 1), which will allow these two QTL to be simultaneously applied for modifying plant morphological architecture.

### Candidate Genes for *QPh.sicau-4B* and *QPh.sicau-6D*

In wheat, several reduced height (*Rht*) genes have been cloned, e.g., *Rht-b1*, *Rht-d1* (Peng et al., 1999), and *Rht18* (Ford et al., 2018). *Rht-B1* encodes a DELLA transcription factor protein, which participates in gibberellin signaling and thus confers the dwarfing trait to the plant (Peng et al., 1999; Sun, 2010). In the *QPh.sicau-4B* interval, 24 genes with low expression were removed (Figure 4), and 47 genes were further annotated using KOBAS 3.0 (Supplementary Table S2), which included *Rht-B1*. The sequence analysis of the *Rht-B1* region revealed that a T for C substitution (C(190)T) converts the codon (CGA) to a translational stop codon (TGA) in CN16 (Figure 5), which corresponded to dwarfing gene *Rht-B1b* (Pearce et al., 2011). Thus, *QPh.sicau-4B* possibly corresponded to dwarfing gene *Rht-B1*.

In the *QPh.sicau-6D* interval, 38 genes with low expression were removed (Figure 4), and 115 genes were further annotated using KOBAS 3.0 (Supplementary Table S2). Among these genes, three have been reported to be involved in plant growth and development and to affect PH in *Arabidopsis* and rice. *YDA* encodes a ubiquitously expressed MAPKK kinase and is sensitive to the hormone signal transduction pathway in dwarf phenotype mutants (Lukowitz et al., 2004). *PAT10* encodes an S-acyltransferase protein, which is critical for development, and the *pat10* mutant demonstrates characteristics such as slow cell expansion and cell division and dwarfism (Zhou et al., 2013). *HUB1* is an important regulatory gene for normal plant development as it is involved in histone H2B monoubiquitination; the *hub1* mutants showed a dwarf phenotype compared with the wild type in *Arabidopsis* and rice (Fleury et al., 2007; Cao et al., 2015). Of these three candidate genes (*TraesCS6D02G227300*, *TraesCS6D02G233000*, and *TraesCS6D02G234900*) for *QPh.sicau-6D*, *TraesCS6D02G233000* has five SNPs in the coding sequence and five SNPs in the non-coding sequence between H461 and CN16 (Figure 5), which led to the substitution of three amino acids (V/A, R/G, T/N). Thus, the gene *TraesCS6D02G233000* might be the candidate gene for further research on *QPh.sicau-6D*.

## Conclusion

In conclusion, two major stable QTL controlling PH were identified in the HCN population across different environments and were validated in the HCM and HMM populations. *QPh.sicau-4B* possibly corresponded to dwarfing gene *Rht-B1*. *QPh.sicau-6D* appears to be a novel QTL for PH, with pleiotropic effects on the SL, TKW, and SC, and thus, *QPh.sicau-6D* is a potential locus worth exploring further for genetic improvement in wheat breeding programs.

## Data Availability Statement

The raw data supporting the conclusions of this article will be made available by the authors, without undue reservation.

## Author Contributions

ZW and HH drafted and revised the manuscript. XJ, YT, YL, FW, and SH conducted phenotype data analysis and contributed to QTL analysis. CL performed the phenotypic evaluation and helped with data analysis. SL and GC helped to draft the manuscript. YXL designed and coordinated this study and revised the manuscript. All authors have read and approved the final manuscript.

## Conflict of Interest

The authors declare that the research was conducted in the absence of any commercial or financial relationships that could be construed as a potential conflict of interest.

## Footnotes

1 http://www.fao.org/faostat/en/#data

2 http://wheat.pw.usda.gov/ggpages/wgc/98/Intro.htm

3 http://www.wheat-expression.com/

## Supplementary Material

Supplementary Figure 1 Frequency distribution of PH in the HCN population.

Supplementary Figure 2 Student’s test the groups carry the alleles from H461 or CN16 at *QPh.sicau-4B* for spikelet number per spike (SN), spike length (SL), spikelet compactness (SC), thousand kernel weight (TKW), anthesis date FT.

Supplementary Figure 3 Student’s test the groups carry the alleles from H461 or CN16 at *QPh.sicau-6D* for spikelet number per spike (SN), spike length (SL), spikelet compactness (SC), thousand kernel weight (TKW), anthesis date FT.

Supplementary Table 1 The primers used in validation population.

Supplementary Table 2 The HC-genes in the *QPh.sicau-4B* interval and *QPh.sicau-6D* interval.

Supplementary Table 3 Comparison of the locations of reported PH quantitative trait loci on chromosome 4B with *QPh.sicau-4B* and chromosome 6D with *QPh.sicau-6D*.
