# Supplementary figures and images for: Identification and Validation of a Novel Major Quantitative Trait Locus for Plant Height in Common Wheat (Triticum aestivum L.)
Source: Front Genet. 2020 Oct 22;11:602495. doi: 10.3389/fgene.2020.602495 (PMC7642865; doi:10.3389/fgene.2020.602495)

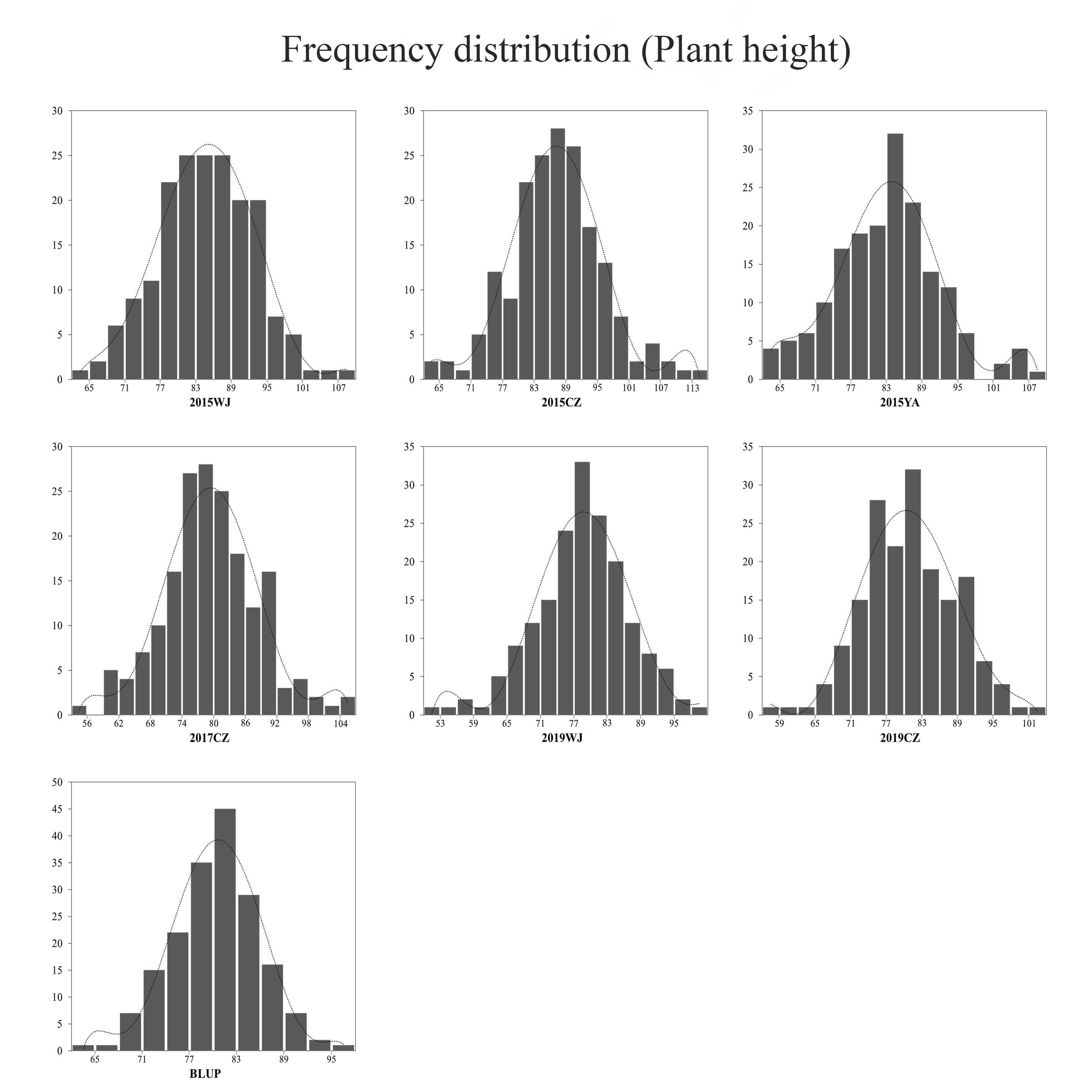

Supplement: Supplementary Figure 1 — Frequency distribution of PH in the HCN population. [file Image_1.PNG]

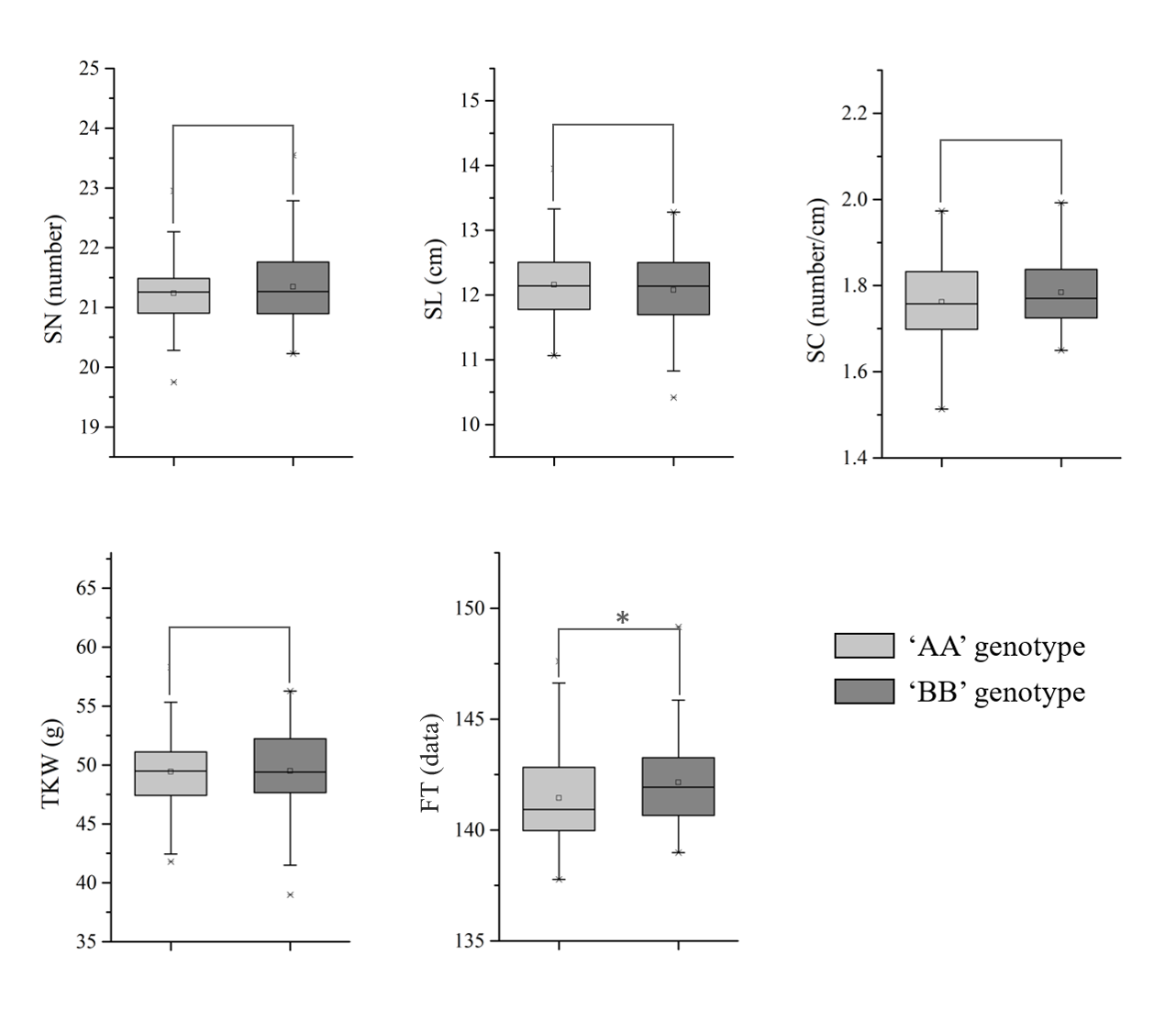

Supplement: Supplementary Figure 2 — Student’s test the groups carry the alleles from H461 or CN16 at QPh.sicau-4B for spikelet number per spike (SN), spike length (SL), spikelet compactness (SC), thousand kernel weight (TKW), anthesis date FT. [file Image_2.PNG]

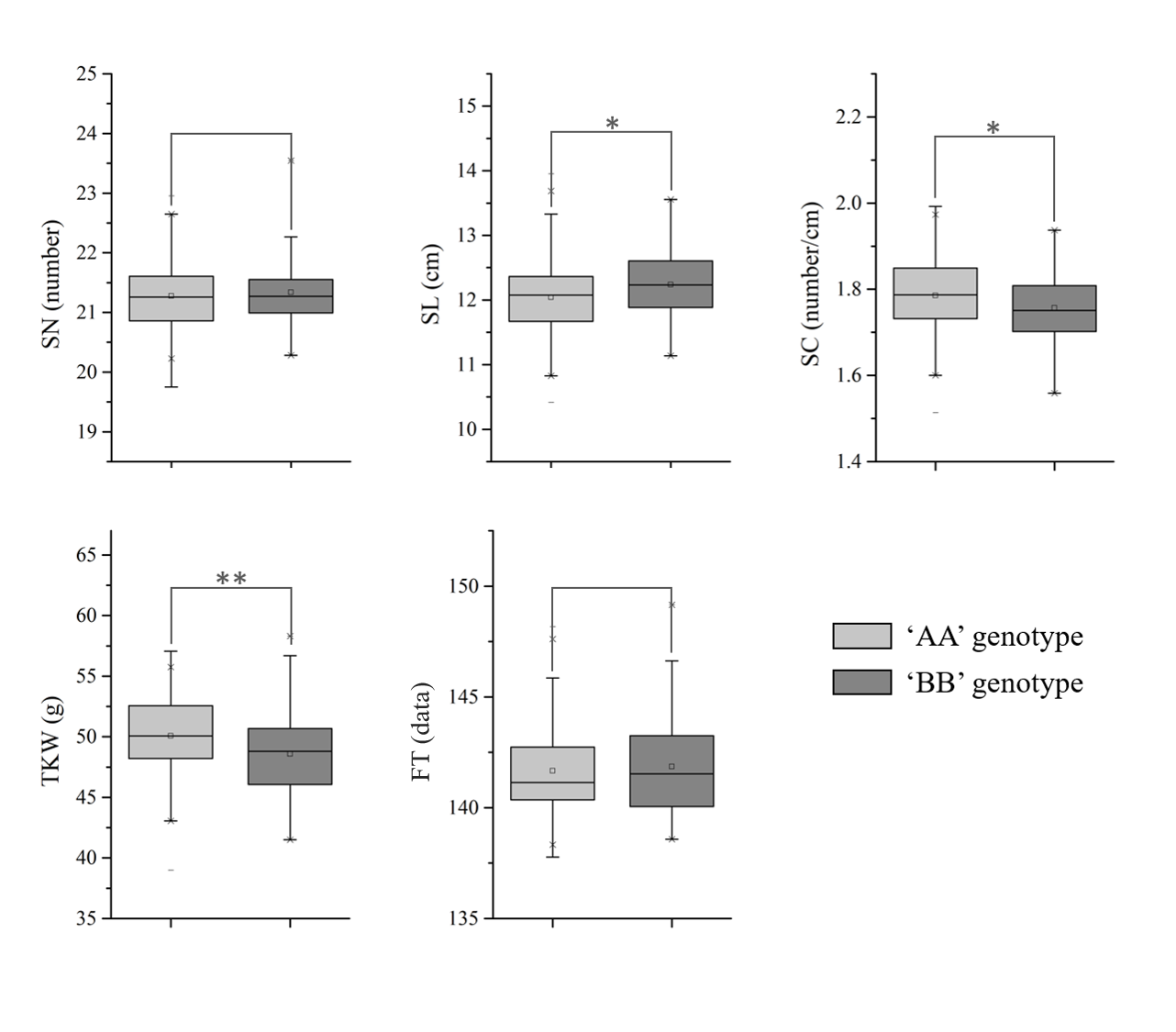

Supplement: Supplementary Figure 3 — Student’s test the groups carry the alleles from H461 or CN16 at QPh.sicau-6D for spikelet number per spike (SN), spike length (SL), spikelet compactness (SC), thousand kernel weight (TKW), anthesis date FT. [file Image_3.PNG]
